# Diagnosis of Ocular Toxocariasis by Serum and Aqueous Humor IgG ELISA

**DOI:** 10.1167/tvst.10.8.33

**Published:** 2021-07-29

**Authors:** Li Huang, Limei Sun, Chengxi Liu, Songshan Li, Ting Zhang, Xiaoling Luo, Xiaoyan Ding

**Affiliations:** 1 State Key Laboratory of Ophthalmology, Zhongshan Ophthalmic Center, Sun Yat-sen University, Guangzhou, China; 2 Department of General Surgery, Peking Union Medical College Hospital, Chinese Academy of Medical Sciences and Peking Union Medical College, Beijing, China

**Keywords:** ocular toxocariasis, diagnostic value, intraocular fluid anti-toxocara IgG, serum anti-toxocara IgG, Goldmann–Witmer coefficient

## Abstract

**Purpose:**

Ocular toxocariasis (OT) is a worldwide ocular parasitic infection and is especially sight-threatening in children. Because of the clinical manifestation diversity, OT has frequently been misdiagnosed. The purpose of this study was to evaluate the diagnostic value of anti-toxocara immunoglobulin G (IgG) in intraocular fluid (IF) and serum in OT.

**Methods:**

IF and serum were collected from patients with clinically diagnosed OT and non-OT uveitis. The level of anti-toxocara IgG was detected by enzyme-linked immunosorbent assay. The data were statistically analyzed in anti-toxocara IgG and the Goldmann–Witmer coefficient (GWC) between groups. The area under the receiver operating characteristic curve (AUC ROC) was performed to assess the diagnostic value of serum and IF anti-toxocara IgG and the GWC.

**Results:**

A total of 290 participants, 128 (44.1%) with OT and 162 (55.9%) with non-OT uveitis, were included in this study. The default serum anti-toxocara IgG cutoff value of 11 U had 72.1% sensitivity and 95.5% specificity. With the optimized cutoff value of 8.2 U, the AUC was 0.886 (95% confidence interval [CI] = 0.830–0.929, *P* < 0.0001), sensitivity increased to 80.2%, and specificity was 94.0%. With an IF anti-toxocara IgG cutoff value of 1.8 U, the AUC was 0.934 (95% CI = 0.892–0.963, *P* < 0.0001), sensitivity was as high as 88.4%, and specificity was 96.4%.

**Conclusions:**

Our study proposes novel diagnostic cutoff values of serum and IF anti-toxocara IgG for OT, which are 8.2 U and 1.8 U, respectively.

**Translational Relevance:**

This study will improve the accuracy of diagnosis in patients with OT.

## Introduction

Ocular toxocariasis (OT), also called ocular larva migrans, is a worldwide parasitic infection that mainly affects pediatric populations, especially in impoverished communities.^1^^–^^4^ OT manifesting as granulomatous uveitis can be classified into four subtypes: peripheral granuloma, posterior pole granuloma, chronic endophthalmitis, and combined type.^2^

OT was initially reported in a globe that had been enucleated due to misdiagnosis as retinoblastoma.^5^ Misdiagnosis as retinoblastoma and subsequent enucleation of the globe compromises the patient's quality of life. However, even with the current understanding of OT, enucleations of globes with OT still occur, even in the most developed countries. Chuah reviewed 26 enucleated eyes diagnosed with retinoblastoma in Singapore and found that one eye (3.5%) with OT had been misdiagnosed.^6^ Shields reviewed 604 enucleated eyes diagnosed with retinoblastoma in Philadelphia, PA, USA, and found that 4% had OT.^7^ Yang et al. evaluated 70 enucleated eyes in Guangdong Province, China, and found one (1.4%) with OT.^8^ Due to the diversity of its clinical manifestations, OT has also been frequently misdiagnosed as Coats disease, persistent hyperplastic primary vitreous, or uveitis with other etiologies.^7^^,^^9^

The gold standard for OT diagnosis is identifying toxocara larvae in biopsy specimens. This is challenging, as it is difficult and risky to obtain a proper specimen for biopsy from the eye.^5^^,^^10^ Currently, diagnosis relies on the typical clinical signs and symptoms, thus depends on the physician's knowledge.^11^ Dana Woodhall et al. elucidated the diagnostic criteria of OT that OT is diagnosed by the identification of clinical signs consistent with disease on ophthalmologic examination, supported by testing for antibody to the toxocara parasite,^12^ as well as Martínez-Pulgarín et al.^13^ In 1986, Genchi assessed the serodiagnosis of ocular toxocariasis, demonstrating that specific immunoglobulin E (IgE) and G (IgG) toxocara antibodies could be used as laboratory evidence of the disease.^14^ However, the interpretation of the required enzyme-linked immunosorbent assay (ELISA) results is not simple. Serum toxocara antibody tested positive in 2 to 18% of an apparently healthy population, suggesting possible past, self-cured infections.^15^ On the other hand, even if serological toxocara antibody is negative, diagnosis of OT cannot be excluded.^16^^–^^19^ Therefore, the detection of anti-toxocara IgG in the intraocular fluid (IF) has been suggested to confirm the diagnosis.^17^ However, to date, a diagnostic cutoff value for IF anti-toxocara IgG has not been reported, and the diagnostic value of the Goldmann–Witmer coefficient (GWC) remains uncertain. For a more objective and precise diagnosis of OT, in this study, we detected the level of specific toxocara antibodies in serum and in IF, to further analyze their diagnostic value.

## Methods

This study was conducted in accordance with the guidelines described in the Declaration of Helsinki and was approved by the Institutional Review Board of the Zhongshan Ophthalmic Center (ZOC), Sun Yat-sen University. Informed consent was obtained from all participants or their guardians prior to the collection of the clinical data and samples.

Two hundred ninety patients, of whom 128 (84 male subjects and 44 female subjects) were clinically confirmed patients with unilateral OT and 162 (80 male subjects and 82 female subjects) patients were clinically confirmed non-OT were included in this retrospective study. The clinical diagnosis of OT was based on the following criteria: (1) typical and characteristic manifestations, including unilateral chorioretinal granuloma in the periphery or the posterior pole,^2^^,^^20^ and (2) exclusion of other ocular diseases, such as ocular toxoplasmosis, sarcoidosis, ocular tuberculosis, and other infectious uveitis. Patients with uncertain diagnosis or a history of ocular surgery or medical treatment were excluded from the study. The non-OT group included patients with a final diagnosis of other etiologies of uveitis or vitreous retinal diseases. The diagnosis was confirmed by two experienced pediatric retina and uveitis ophthalmologists (authors XD and LS). The participants were referred to ZOC between March 2016 and December 2019.

The demographic and clinical data were collected consisting of age, gender, age of presentation, complaints, and family history. A complete ophthalmic examination, including best corrected visual acuity (BCVA) measurement, intraocular pressure, slit-lamp examination, and fundus biomicroscopy, was performed on each participant. Paired IF from the aqueous humor (AH) and serum samples from each participant were collected. The sample collections were done before initiation of any treatment. The anti-toxocara IgG levels in the samples were determined using an ELISA kit (toxocara *canis* IgG ELISA; IBL International, Inc., Germany), which contains micro test wells coated with synthetic glycopeptides that are immunologically similar to excretory-secretory antigens from *T. canis* larvae. The antibody level unit (U) was calculated as (sample absorbance × 10)/cutoff value. The samples were considered positive if exceeding the default cutoff value of 11 U recommended by the manufacturer. The GWC was calculated as (specific IgG in IF/specific IgG in serum)/ (total IgG in IF/total IgG in serum). The assay was performed according to the manufacturer's instructions.

The data were analyzed using IBM SPSS Statistics version 22.0. The Kolmogorov–Smirnov test was used to test the normality of the quantitative data. When the data followed a normal distribution (*P* > 0.05), the mean and standard deviation (SD) were used to describe them, and the independent sample *t*-test was used to compare the population mean of the groups. The association between qualitative variables was assessed using odds ratios (ORs) with 95% confidence intervals (CIs) by logistic regression analysis. The receiver operator characteristic (ROC) curve was used to test the diagnostic values of IF and serum anti-toxocara IgG and of the GWC. The Youden index (YI) was calculated as (sensitivity + specificity − 1). The optimal cutoff point was determined from the ROC curve. A Z-test was performed in the area under the curve (AUC) to test the differences between diagnostic values.

## Results

### Demographic Data

**Figure 1.**
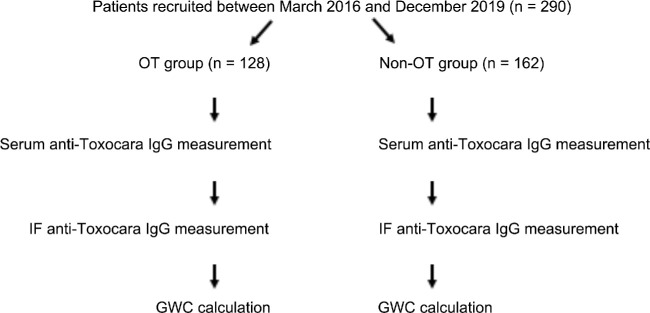
Schematic overview of patient selection.

A clinical diagnostic trial was conducted in 290 patients, of whom 128 (84 male subjects and 44 female subjects) patients were clinically confirmed with unilateral OT and 162 (80 male subjects and 82 female subjects) were clinically confirmed non-OT patients (Fig. 1). The mean age of the patients in the OT group was 10.50 ± 8.62 years, and that of the patients in the non-OT group was 13.16 ± 9.32 years. There were 111 children (86.7%) in the OT group and 116 (71.6%) in the non-OT group. The patients’ demographic data are summarized in Table 1.

**Table 1.** Demographics of the OT and Non-OT Groups

Variable	OT^a^	Non-OT	*P* Value
Patients: number (n, %)	128 (44.1)	162 (55.9)	_
Male (n, %)	84 (65.4)	80 (49.4)	_
Female (n, %)	44 (34.4)	82 (50.6)	_
Children (<18 y old)	111 (86.7)	116 (71.6)	_
Mean age (y ± SD)	10.50 ± 8.62	13.16 ± 9.32	_
OD^b^ (n, %)	66 (51.6)	82 (50.6)	_
OS^c^ (n, %)	62 (48.4)	80 (49.4)	_
IF^d^ anti-toxocara IgG (U ± SD)	25.15 ± 19.78	1.14 ± 0.38	**<0.001**
IF total IgG (U ± SD)	62175.15 ± 307837.88	935.74 ± 2335.90	0.461
Serum anti-toxocara IgG (U ± SD)	22.55 ± 15.42	4.37 ± 4.71	**<0.001**
Serum total IgG (U ± SD)	26979.94 ± 36087.35	34912.94 ± 39773.47	**<0.001**
GWC^e^	103.28 ± 262.21	47.03 ± 54.24	0.435

a Ocular toxocariasis.

b Right eye.

c Left eye.

d Intraocular fluid.

e Goldmann–Witmer coefficient.

After the Kolmogorov–Smirnov test, the IF and serum anti-toxocara IgG and the GWC followed a normal distribution (*P* = 0.287, *P* = 0.195, and *P* = 0.350, respectively). The concentrations of IF and serum anti-toxocara IgG in the OT group were higher than those in the non-OT group (*P* < 0.001). The total serum IgG in the OT group was lower than that in the non-OT group (*P* < 0.001). On the other hand, no significant difference between the two groups was observed in terms of total IF IgG (*P* = 0.461) and GWC (*P* = 0.435; see Table 1).

### Diagnostic Performance of Serum Anti-Toxocara IgG With the Manufacturer-Recommended Cutoff Value

Serum anti-toxocara IgG was considered positive when the value was higher than 11 U by the manufacturer. In this study, we first used the same value to analyze our data. The recommended serum anti-toxocara IgG cutoff value of 11 U yielded a YI value of 0.676, 72.1% sensitivity, 95.5% specificity, 96.4% positive predictive value, and 66.4% negative predictive value. Considering the low sensitivity and negative predictive value of the recommended cutoff value of 11 U, we aimed to optimize the cutoff value for OT diagnosis.

### Receiver Operating Characteristic Curve to Optimize the Cutoff Value for Serum Anti-Toxocara IgG

**Figure 2.**
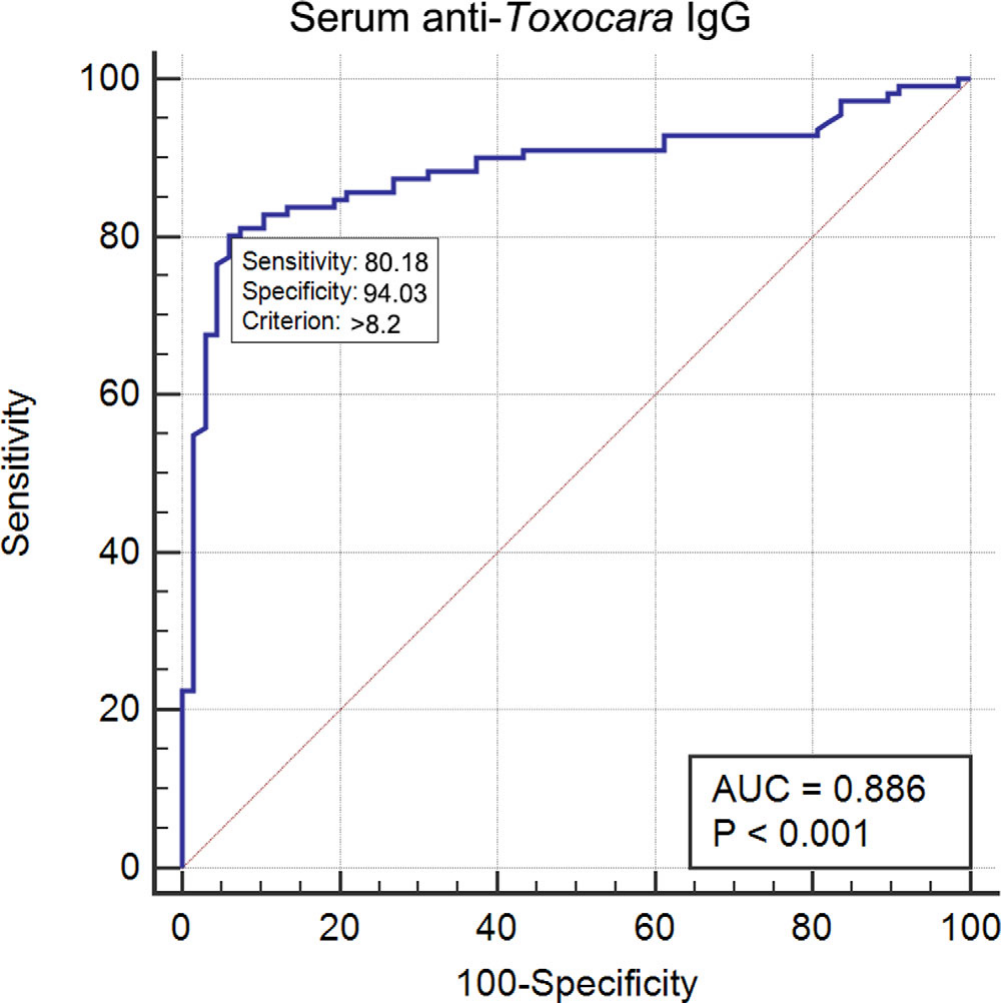
ROC analyses of the diagnostic value of serum anti-toxocara IgG for OT as a reference standard, as evidenced via clinical examination. The best cutoff value for definite OT diagnosis was found at 8.2 U, which yielded 80.2% sensitivity and 94.0% specificity.

To redefine the cutoff value for serum anti-toxocara IgG, we calculated the area under the ROC curve. The results showed that the AUC of serum anti-toxocara IgG was 0.886 (95% CI = 0.830–0.929, *P* < 0.0001). A cutoff value of 8.2 U yielded the highest YI (0.742), 80.2% sensitivity, and 94.0% specificity (Table 2, Fig. 2). Its positive predictive value was 94.7%, and its negative predictive value was 73.8%. Using this novel cutoff value, logistic regression showed that higher serum anti-toxocara IgG levels were observed more frequently in the OT than in the non-OT group (*P* = 0.001, OR = 4.30, 95% CI = 3.46–5.79; see Table 2).

**Table 2.** Statistic of ROC in IgG Anti-Toxocara of Serum, IF, and GWC

	Serum Anti-Toxocara IgG	IF^e^ Anti-Toxocara IgG	GWC^f^
Sample size (total)	178	214	88
OT^a^	111 (62.36%)	103 (48.13%)	74 (84.09%)
Non-OT	67 (37.64%)	111 (51.87%)	14 (15.91%)
AUC^b^	0.886	0.934	0.507
SE^c^	0.026	0.022	0.078
95% CI^d^	0.830 to 0.929	0.892 to 0.963	0.398 to 0.616
*P* value	**<0.0001**	**<0.0001**	0.926
Youden index	0.742	0.848	0.127
Associated criterion	>8.2	>1.8	>75.5
Sensitivity (%)	80.18	88.35	27.03
Specificity (%)	94.03	96.4	85.71
Positive predictive value (%)	94.68	94.79	91.3
Negative predictive value (%)	73.81	89.83	17.19
Odds ratio	4.30	5.31	_
95% CI of odds ratio	3.46 to 5.79	4.40 to 7.18	_

a Ocular toxocariasis.

b Area under the ROC curve.

c Standard error.

d Confidence interval.

e Intraocular fluid.

f Goldmann–Witmer coefficient.

Two (3.0%) patients who showed positive serum anti-toxocara IgG (>8.2 U) were not diagnosed with OT. These false positive patients had borderline serum anti-toxocara IgG levels (12.48 U and 8.22 U, respectively). A 24-year-old female patient whose serum anti-toxocara lgG was 12.48 U presented with unilateral retinal fold, tractional retinal detachment, posterior synechia, cataract, and uveitis. She was eventually diagnosed with Stickler syndrome with a *COL11A1* heterozygous mutation. A 12-year-old female patient with a serum anti-toxocara IgG value of 8.22 U presented with unilateral intraocular inflammation with stratified vitreous and was considered a possible OT patient. Conversely, 19 (17.1%) false negative patients, that is, patients with negative serum anti-toxocara lgG values (<8.2 U), were eventually diagnosed with OT. Twelve of them (63.2%) had advanced or end-stage OT manifesting as retinal folds (6 eyes), tractional retinal detachment (3 eyes), cataract and posterior synechia (4 eyes), and esotropia (4 eyes), and their median BCVA was 2.2 by logarithm of the minimum angle of resolution (logMAR). In these patients, the average interval between onset and diagnosis was 6.25 ± 2.53 months (range = 4–12, median = 5.5 months).

### Receiver Operating Characteristic Curve for IF Anti-Toxocara IgG

**Figure 3.**
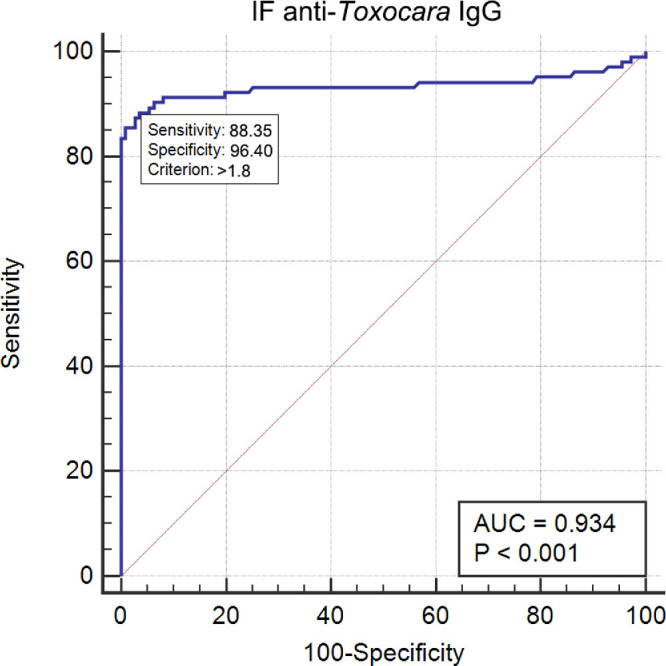
ROC analyses of the diagnostic value of IF anti-toxocara IgG for OT as a reference standard, as evidenced via clinical examination. The best cutoff value for definite OT diagnosis was found at 1.8 U, which yielded 88.4% sensitivity and 96.4% specificity.

**Figure 4.**
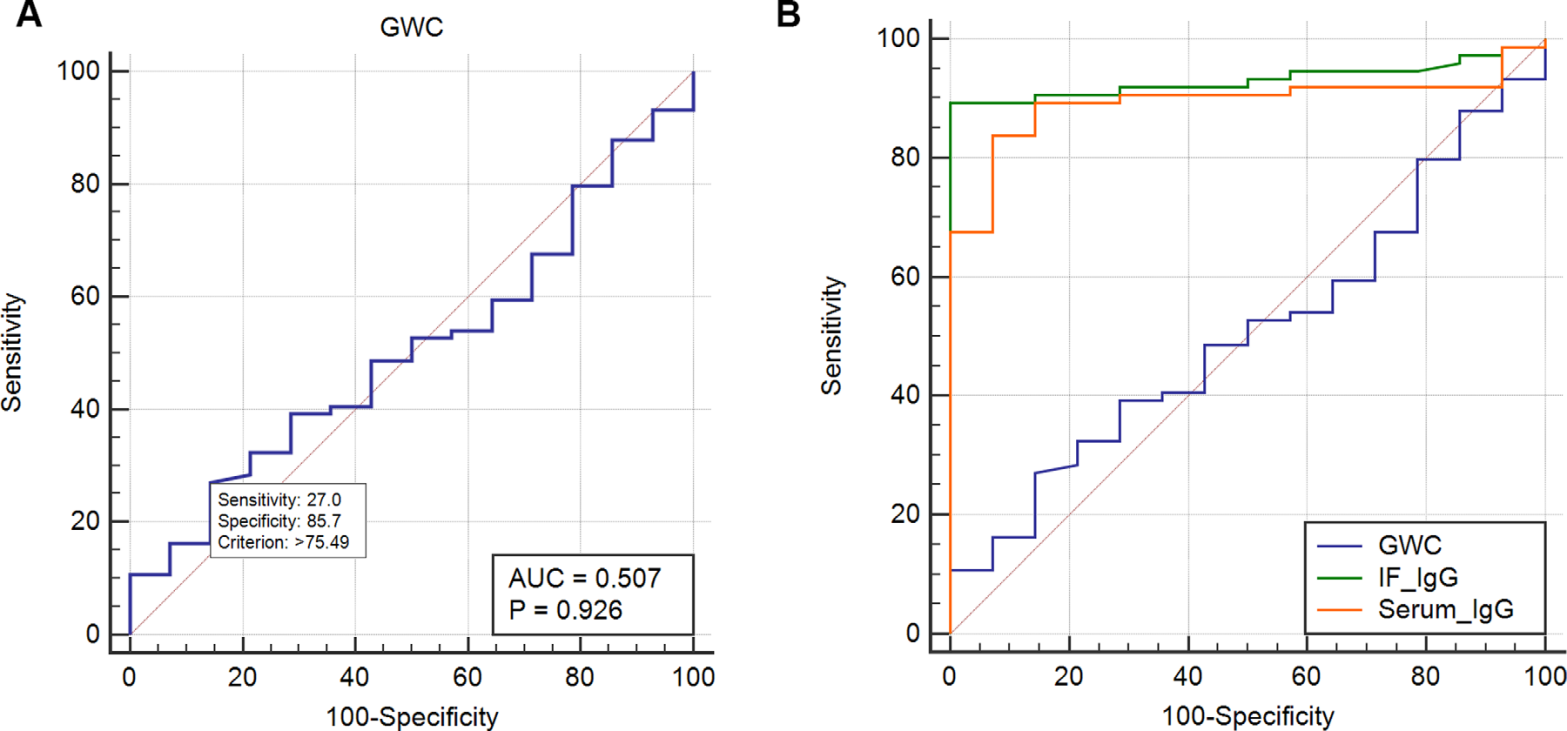
(**A**) ROC analyses of the diagnostic performance of the GWC. The best cutoff value for definite OT diagnosis was found at 75.5, which yielded 27.0% sensitivity and 85.7% specificity (*P* = 0.926). (**B**) Comparison between the AUC of serum anti-toxocara IgG, IF anti-toxocara IgG, and the GWC.

The AUC of IF anti-toxocara IgG was 0.934 (95% CI = 0.892−0.963, *P* < 0.0001). A cutoff value of 1.8 U yielded a YI of 0.848, 88.4% sensitivity, and 96.4% specificity (see Table 2, Fig. 3). Its positive predictive value was 94.8%, and its negative predictive value was 89.8%. At this cutoff value, higher IF anti-toxocara IgG concentrations were observed more frequently in the OT than in the non-OT group (*P* = 0.001, OR = 5.31, 95% CI = 4.40−7.18; see Table 2). A pairwise comparison of ROC curves showed no significant difference between the serum and the IF anti-toxocara IgG. Their AUC difference was 0.038 (*P* = 0.3436, Table 3, Fig. 4B).

**Table 3.** Comparison of ROC

Variable	Difference Between AUC	SE^a^	95% CI^b^^†^	*P* Value
GWC^c^: IF^d^, anti-toxocara IgG	0.424	0.078	0.271 to 0.576	**<0.0001**
GWC: Serum anti-toxocara IgG	0.384	0.096	0.197 to 0.574	**0.0001**
IF anti-toxocara IgG: Serum anti-toxocara IgG	0.038	0.04	−0.041 to 0.117	0.3436

a Standard error.

b Confidence interval.

c Goldmann–Witmer coefficient.

d Intraocular fluid.

IF anti-toxocara IgG levels exceeding 1.8 U were detected in five (4.5%) patients in the non-OT group, all of whom had borderline serum anti-toxocara IgG levels. A 16-year-old male patient with unilateral uveitis exhibiting massive subretinal yellowish-white exudation in fundus photography was eventually diagnosed with Coats disease because of a general dilatation of his capillaries revealed by fundus fluorescein angiography (FFA). A 29-year-old male patient showed unilateral intraocular inflammation with peripheral granuloma with no leakage in FFA and seropositive aspergillus antigen. Three cases presented with unilateral intraocular inflammation with vitreous opacity. Eleven (10.7%) patients in the OT group had IF anti-toxocara IgG levels below 1.8 U. Four of them (36.4%), whose median BCVA was logMAR 3, had advanced or end-stage OT manifesting as retinal folds (3 eyes), tractional retinal detachment (1 eye), cataract and posterior synechia (1 eye), and dense vitreous strands (2 eyes). In these patients, the average interval between onset and diagnosis was 14.25 ± 14.93 months (range = 4–36, median = 8.5 months). Four (36.4%) patients had peripheral (1 eye) or posterior pole granuloma (3 eyes) in the fundus without leakage in FFA. The remaining three patients with negative serum and IF anti-toxocara IgG had advanced or end-stage OT.

### Diagnostic Performance of the Goldmann–Witmer Coefficient

**Figure 5.**
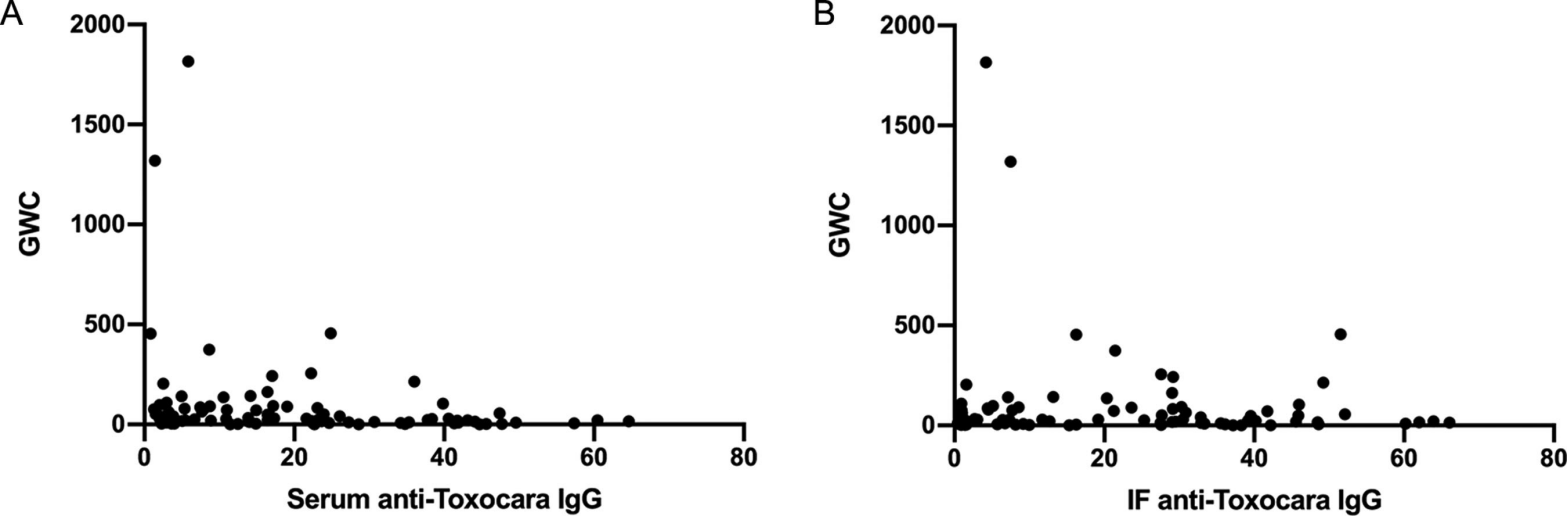
(**A**) Correlation between serum anti-toxocara IgG and GWC (r = −0.20, *P* = 0.06). (**B**) Correlation between IF anti-toxocara IgG and GWC (r = −0.08, *P* = 0.47).

The GWC was calculated in 88 patients. The AUC evaluation showed that the overall GWC had poor diagnostic performance for OT, with a value of 0.507 (95% CI = 0.398–0.616, *P* = 0.926; see Table 2, Fig. 4A). The GWC was further analyzed in the IF anti-toxocara IgG-positive (>1.8 U) and IgG-negative (<1.8 U) groups. In 59 of the 65 (90.8%) patients with IgG-positive disease and in 21 of the 23 (91.0%) IgG-negative patients, the GWC was >3, with no significant difference between the 2 groups (*P* = 0.939). The scatterplots shown in Figure 5 revealed no correlation between serum/IF anti-toxocara IgG and GWC. However, there was significant difference if we were only analyzing the GWC of IF anti-toxocara IgG-positive (>1.8 U) or serum anti-toxocara IgG-positive (>8.2 U) between OT and non-OT groups (*P* < 0.0001; Supplementary Table S1). The results indicated that only when the serum or IF anti-toxocara IgG is positive, positive GWC (>3) has the referential values in diagnosing ocular toxocariasis.

### Validation of the Novel Cutoff Values in Serum/IF Anti-toxocara IgG in an Independent Group

Twenty-eight novel independent sample pairs (IF and serum), 18 from patients with OT and 10 from non-OT patients, were used to validate the cutoff values of 1.8 U (IF) and 8.2 U (serum). The former value showed 88.9% sensitivity and 90.0% specificity, and the latter showed 94.4% sensitivity of and 80.00% specificity (Table 4).

**Table 4.** Test the Cutoff Value in Newly Collected Samples

Variable		OT^a^ (18)	Non-OT (10)	Total (28)	*P* Value
IF^b^ anti-toxocara IgG	Positive, n (%)	16 (88.89)	1 (10.00)	17 (60.71)	**<0.0001**
	Negative, n (%)	2 (11.11)	9 (90.00)	11 (39.29)	
Serum anti-toxocara IgG	Positive, n (%)	17 (94.44)	2 (20.00)	19 (67.86)	**<0.0001**
	Negative, n (%)	1 (5.56)	8 (80.00)	9 (32.14)	

a Ocular toxocariasis.

b Intraocular fluid.

## Discussion

Toxocariasis belongs in a group of diseases known as neglected parasitic infections. These diseases are targeted by the US Centers for Disease Control and Prevention for public health action. They are considered neglected because relatively little attention has been paid to their monitoring, prevention, and/or treatment. OT shares some clinical features with uveitis with other etiologies, vitreoretinopathy, and retinoblastoma.^7^^,^^21^ OT diagnosis can be hindered by the atypical clinical signs and limitations in the physician's experience.^11^ In China, like in other countries, the clinical awareness of the disease is insufficient.^22^ Thus, in most scenarios, clinical characteristics cannot serve as a standard diagnostic tool; therefore, laboratory examinations are very helpful.

In the absence of parasitological evidence and because the toxocara life cycle is not completed in humans, the immune response becomes a helpful diagnostic tool. Previous studies have reported the importance of anti-toxocara IgG serology for the diagnosis of OT.^19^^,^^23^ Other studies, however, have suggested that IgG anti-toxocara antibodies can often be undetectable in the sera of patients with OT, and consequently serologic screening is not informative for the diagnosis.^22^^,^^24^^–^^26^ The sensitivity and specificity of serum ELISA have been reported as approximately 90%.^23^ However, OT cannot be excluded on the basis of negative results, and positive results cannot lead to a secure diagnosis, as they may be due to occult asymptomatic systemic toxocara infections. Therefore, we conducted a relatively large-scale study to determine the diagnostic value of serum anti-toxocara IgG ELISA in patients with OT, which could be valuable for the interpretation of assay results and for differential diagnosis.

Previous studies have reported different serum anti-toxocara IgG results.^19^^,^^23^^,^^25^ Bae et al., using a TCLA ELISA kit that detected IgG antibody titers specific to the toxocara *canis* larva crude antigen (Korea, not commercially available), evaluated serum anti-toxocara IgG in 278 patients with uveitis, including 71 patients with OT, setting the cutoff value at 0.250 titers and reporting 91.5% sensitivity and 91.0% specificity.^23^ Abd El-Aal et al. assessed serum anti-toxocara IgG in 30 patients with OT and 82 non-OT patients and found that at a cutoff value of 0.258 titers, the sensitivity and specificity of IgG ELISA were 93.3% and 100%, respectively.^19^ In this study, we confirmed the diagnostic value of serum anti-toxocara IgG using the units, which was (sample absorbance × 10)/cutoff value. A value of 8.2 U yielded moderate sensitivity (80.2%) and high specificity (94.0%).

Specific antibodies can also be detected in the AH and vitreous humor (VH). However, the diagnostic value of anti-toxocara IgG in the IF has rarely been reported, and in most cases, by case reports. Glickman et al. described a case with serologically proven visceral toxocariasis where toxocara-specific antibodies were also detected in the AH.^27^ Inchauspe et al. reported six OT cases with negative serological anti-toxocara IgG subsequently confirmed by positive vitreous anti-toxocara IgG.^17^ To our knowledge, our study was the first to focus on the cutoff value of IF anti-toxocara IgG. We found that IF anti-toxocara IgG is an even better diagnostic tool for OT than serum anti-toxocara IgG. A cutoff value of 1.8 U yielded 88.4% sensitivity and 96.4% specificity. Its positive predictive value was 94.8%, and its negative predictive value was 89.8%.

Intraocular production of toxocara antibodies can be assessed by comparing serum and AH samples obtained from the same patient and calculating the GWC. Few studies have included the GWC,^28^ which is designed to exclude false positive cases, to determine whether these specific antibodies are produced in the eye or infiltrate it from the serum.^24^ The GWC should only be considered in IF IgG-positive cases. It has been used not only in OT but also in other ocular diseases.^29^^–^^34^ Robert-Gangneux tested the GWC for biological diagnosis of toxoplasmic retinochoroiditis with 53% sensitivity; when using the GWC combined with immunoblotting, sensitivity increased to 71%.^29^ Similar results were reported by Fekkar and Mathis, who combined the GWC with other diagnostic tools, reporting significantly higher sensitivity than when using the GWC alone.^30^^,^^32^ The GWC has also been used for the diagnosis of cytomegalovirus, herpes simplex virus, and varicella zoster virus infection of the ocular.^33^ However, to date, there has been no systematic evaluation of the use of the GWC for the immunological diagnosis of OT. Wang evaluated the GWC in the immunological diagnosis of OT, indicating that it was more accurate than the evaluation of specific anti-*T. canis* IgG in IF and suggesting reference values.^35^ However, because in our study there was no significant difference in the AUC area between the OT and non-OT groups, we did not find sufficient evidence to confirm the diagnostic efficiency of the GWC. A possible reason is the relatively low rate of serum positivity in our control patients with uveitis.

The limitations of this study should be taken into consideration when interpreting our data. First, this was a retrospective study with a limited sample size due to the rarity of OT, which makes it difficult to recruit a large cohort. Because this is a retrospective study, the gender was mismatched, and a number of patients did not detect the total IgG in serum making it unable to calculate the GWC. Second, as the study was conducted in a tertiary referral institute for pediatric retinal diseases, referral bias cannot be excluded. Further studies with more cases in various cohorts are warranted to confirm our findings. Third, as the ELISA used in our study was immunologically *T. canis*–specific, it may not be appropriate for *T. cati* and might lead to an underdiagnosis of patients with OT caused by this species. Fourth, Rahmah Noordin et al. mentioned that specificity could be improved by measuring Ig G4 subtypes,^36^ however, IgG subtypes were not detected in this study.

In summary, although we cannot confirm the diagnostic value of the GWC, our study confirms the diagnostic value of both serum and IF anti-toxocara IgG for the diagnosis of OT. We propose a novel serum anti-toxocara IgG cutoff value of 8.2 U instead of the default value of 11 U. Finally, we suggest for the first time an IF anti-toxocara IgG cutoff value of 1.8 U.

## Supplementary Material

Supplement 1
