# Supplementary material for: Diagnosis of Ocular Toxocariasis by Serum and Aqueous Humor IgG ELISA
Source: Transl Vis Sci Technol. 2021 Jul 29;10(8):33. doi: 10.1167/tvst.10.8.33 (PMC8322715; doi:10.1167/tvst.10.8.33)
Supplement: Supplement 1 [file tvst-10-8-33_s001.pdf]

Supplementary Table 1. Laboratory data of serum, IF anti-*Toxocara* IgG and GWC

Variable	% of OT patients	% of controls	P value
Positive in serum	75.60%	0%	P<0.0001
Positive in IF	90.50%	0%	P<0.0001
Positive in IF confirmed by GWC	97.01%	0%	P<0.0001
Positive in serum confirmed by GWC	89.29%	0%	P<0.0001
